# Computed Tomography Angiography (CTA) in Selected Scenarios with Risk of Possible False-Positive or False-Negative Conclusions in Diagnosing Brain Death

**DOI:** 10.3390/life12101551

**Published:** 2022-10-06

**Authors:** Gerhard Schwarz, Maximilian Errath, Placido Argüelles Delgado, Ulrike Wießpeiner, Henrika Voit-Augustin, Robert Grims, Friedrich Kaltenböck, Eva Maria Kober, Andreas Schöpfer, Gottfried Fuchs

**Affiliations:** 1 Department of Anaesthesiology and Intensive Care Medicine, Medical University of Graz, A-8036 Graz, Austria; 2 Institute for Medical Informatics, Statistics and Documentation, Medical University of Graz, A-8036 Graz, Austria; 3 Division of Neuroradiology, Vascular and Interventional Radiology, Department of Radiology, Medical University of Graz, A-8036 Graz, Austria; 4 Department of Dermatology, Medical University of Graz, A-8036 Graz, Austria; 5 Division of Anaesthesiology and Intensive Care Medicine, LKH Feldbach-Fürstenfeld, A-8330 Feldbach, Austria

**Keywords:** brain death, computed tomography angiography, computed tomography perfusion, cerebral blood perfusion, neuroimaging, expert system, transplantation medicine, intensive care medicine

## Abstract

It is widely accepted that brain death (BD) is a diagnosis based on clinical examination. However, false-positive and false-negative evaluation results may be serious limitations. Ancillary tests are used when there is uncertainty about the reliability of the neurologic examination. Computed tomography angiography (CTA) is an ancillary test that tends to have the lowest false-positive rates. However, there are various influencing factors that can have an unfavorable effect on the validity of the examination method. There are inconsistent protocols regarding the evaluation criteria such as scoring systems. Among the most widely used different scoring systems the 4-point CTA-scoring system has been accepted as the most reliable method. Appropriate timing and/or Doppler pre-testing could reduce the number of possible premature examinations and increase the sensitivity of CTA in diagnosing cerebral circulatory arrest (CCA). In some cases of inconclusive CTA, the whole brain computed tomography perfusion (CTP) could be a crucial adjunct. Due to the increasing significance of CTA/CTP in determining BD, the methodology (including benefits and limitations) should also be conveyed via innovative electronic training tools, such as the BRAINDEXweb teaching tool based on an expert system.

## 1. Introduction

The determination of brain death (BD), also denoted as death by neurologic criteria (DNC) (in Germany as irreversible brain function failure), is one of the most responsible challenges in medical diagnostics, because it has not only medical, but also legal, philosophical, ethical, psychological, socio-cultural or anthroposophical implications. If all the criteria for the diagnosis of BD are met, then the findings of irreversible loss of all brain functions lead to an “end-of-life” decision and also provide the legal prerequisite for a possible organ donation.

It is widely accepted that BD is a diagnosis based on clinical examination. However, potential confounders are manifold and are grouped into general categories including neurological diseases/lesions, hemodynamics (hypotonia) and body temperature (hypothermia) [1], metabolic disturbances, toxicities, sedation effects and other drug effects [2]. There are several confounding factors that may mimic or partially mimic the clinical aspect of brain death and lead to false-positive examination results [3,4,5,6,7,8,9,10,11,12,13,14,15]. In other words, the clinical tests or signs suggest brain death, but the patient is not [16]. In contrast, false-negative clinical signs may also be possible, inducing doubt about the existence of brain death, even though brain death is present [17,18,19,20,21,22]. Therefore, knowledge of the etiology of coma and possible clinical aspects of BD is essential.

This narrative review is based on the research question of how neuroimaging techniques such as CTA and CTP are currently used as ancillary tests in determining BD. We aim to present the role of confirmatory testing in general and in various scenarios with the risk of possible false-negative or false-positive assessment results. In addition, we describe the current status of computed tomography angiography (CTA) scoring systems, the status of computed tomography perfusion (CTP), and measures to ensure the validity of CTA/CTP. Beyond that, we would like to encourage developers of teaching and learning systems to communicate developments in neuroimaging and other facts via innovative interactive electronic training/learning tools for the diagnosis of BD and present an example of how an expert system can be designed for this particular purpose.

**Figure 1:**
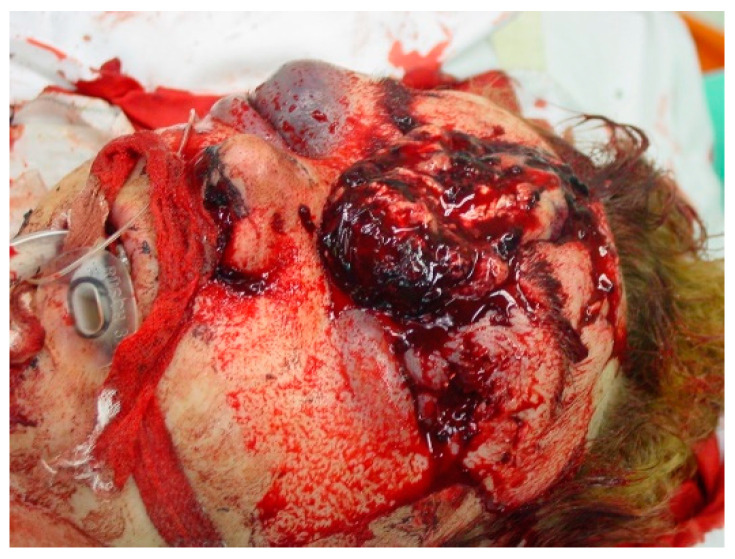
Unresponsive comatose state in severe craniofacial trauma impeding BD diagnosis.

Although complete clinical examination (coma assessment, brainstem reflex testing, evidence of apnea) and conclusive evidence of irreversibility lead to the diagnosis of BD in the vast majority of cases, additional tests (confirmatory tests) play an important role in the diagnostic process. They are used to prove the irreversibility of BD, especially when unusual constellations are present that call into question the examination of the characteristic clinical symptoms due to confounding factors. Therefore, these tests are referred when uncertainty exists about the reliability of parts of the neurologic examination (e.g., a condition that would preclude performance of a complete brainstem reflex test, such as severe facial trauma or swelling (Figure 1) or when a cervical spine lesion [7,8] compromises the test validity and/or the patient’s safety and when the apnea test cannot be performed [1].

Additionally, in some official protocols, ancillary tests are used to shorten the duration of a given observation period [15], especially in unstable patients [23]. Conversely, it should be noted that in some specific constellations ancillary tests may delay the diagnosis of BD and cause damage to the organ donation target by causing confusion by false-negative or false-positive findings [24], if there is no adequate expertise in the interpretation of each of the confirmatory tests.

In 14 of 28 European countries, one or more ancillary or confirmatory tests are required before BD can be diagnosed [25]. Inconsistency in guidelines regarding ancillary tests is present even in the three German-speaking European countries. For example, the current Austrian recommendations for the determination of BD [26] require that the EEG is requested in every case, while CTA or transcranial Doppler sonography/color-coded duplex sonography (TCD/CCDS) are optional, but, catheter-based cerebral angiography is not permitted. In the medical-ethical guidelines on the determination of death with regard to organ transplantation and preparation for organ donation in Switzerland [27], only methods to detect cerebral blood flow are listed as additional technical examinations, such as Doppler sonography, CT angiography, digital subtraction angiography, and magnetic resonance imaging. In Germany [28], evidence of irreversibility of brain function loss can be ancillary based on an isoelectric EEG or extinction of intracerebral components of brainstem auditory evoked potentials (BAEP) or early cortical (N20 component) and high cervical somatosensory evoked potentials (SEP) or detection of cerebral circulatory arrest by TCD/CCDS, cerebral perfusion scintigraphy or CT angiography. For an overview of ancillary examinations see Table 1, which is based on the three official recommendations described [26,27,28] and articles by various authors [15,21,29,30,31,32,33,34,35]. These three examples show that several supplementary tests are equally allowed in the national recommendations, which may lead to misinterpretations of relevance between the different tests (especially, if the supplemental guidance on the limitations and scope of specific testing procedures in the respective official guidelines is over-read in detail and/or not interpreted correctly by users). However, intensivists ordering supplementary tests should be aware of possible discrepancies between the results of the tests and the clinical symptoms of the patients in individual cases. They should, therefore, critically question the causes by consulting experts in the respective fields.

## 2. Ancillary Tests for Determination of Cerebral Blood Perfusion Arrest

An ideal ancillary test should not give false-positive results (i.e., a declaration of death when there is no brain death). In other words, 100% specificity should be the goal in this particular clinical situation. According to the statements of the “World Brain Death Project” [1] concerning blood perfusion tests, the sensitivity varies mainly in a range of 52–100% while specificity is close to 100%, but there is the remark that specificity is assumed on the basis of experimental data and should be therefore interpreted with caution given the limitation of studies that reported only on clinically confirmed BD/DNC [29].

Evidence of cerebral perfusion arrest is based on the reasonable assumption that a tissue that is not perfused is no longer alive. Therefore, ancillary tests showing circulatory cessation of the brain are considered suitable methods. Cerebral angiography (digital subtraction angiography—DSA, using catheters, X-ray imaging guidance and contrast material to examine blood vessels in the brain) has been considered the reference for the confirmation of cerebral circulatory arrest (CCA), but these methods have significant disadvantages such as invasiveness, technical complexity or limited infrastructural accessibility [36]. As these tests should only be used as part of the patient’s treatment for their benefit (presupposing the possibility of therapeutic consequences), these methods have been removed from the guidelines for determining BD in some countries [37]. For this purpose, the cerebral perfusion scintigraphy as well as the transcranial Doppler sonography (TCD)/color-coded duplex sonography (CCDS) were introduced [26,28]. In a further step, CTA was considered to be a promising ancillary test by some official institutions [26,28], as the method is characterized by minimal-invasiveness, ease of access in most hospitals, lower operator dependence, and faster execution [38]. New-generation multi-slice CT scanners are suitable to visualize vasculature and perfusion of the whole brain with a single intravenous injection of iodinated contrast medium to verify cerebral perfusion stop. National guidelines introduced CTA in several countries e.g., Austria, Canada, Croatia, France, Germany, Netherlands, Poland or Switzerland [26,29,39,40].

## 3. CTA-Diagnosing Management for BD Determination

CTA is considered the most appropriate complementary test to clarify an unclear BD diagnosis, as this ancillary test tends to have the lowest false-positive rates [41,42]. In a clinical study a group of 82 BD-patients validating CTA against DSA a sensitivity of 96.3% of CTA was shown [30]. This study is one of the growing number of publications supporting the inclusion of CTA as accepted ancillary tests in BD-diagnosing recommendations [41]. However, there is currently no international consensus regarding CTA-rating. The differences between the applied protocols are e.g., arterial or venous phase scan duration, scoring systems, evaluation of blood flow phases, specific vessels, and the number of vessels [43]. According to the literature of the last quinquennial time frame, the choice of the appropriate scoring systems plays an important role for the validity of CTA in the context of BD diagnosis. The most frequently used scoring systems are the 10-, 7- and 4-points scores [29]. The interobserver agreement was high for all scales in previous studies [36,43]. However, comparative studies of these scoring systems showed considerable differences in sensitivity [44].

Shahin & Pekçevik, Y. (2015) [43] found a sensitivity obtained for 10-and 7-point scales was 52% and 64%, respectively. Examples of the limited value of these two scales can be shown especially in constellations with compromised skull integrity of various causes such as trauma or operative intracranial pressure relief in patients with clinical signs of brain death.

**Figure 2:**
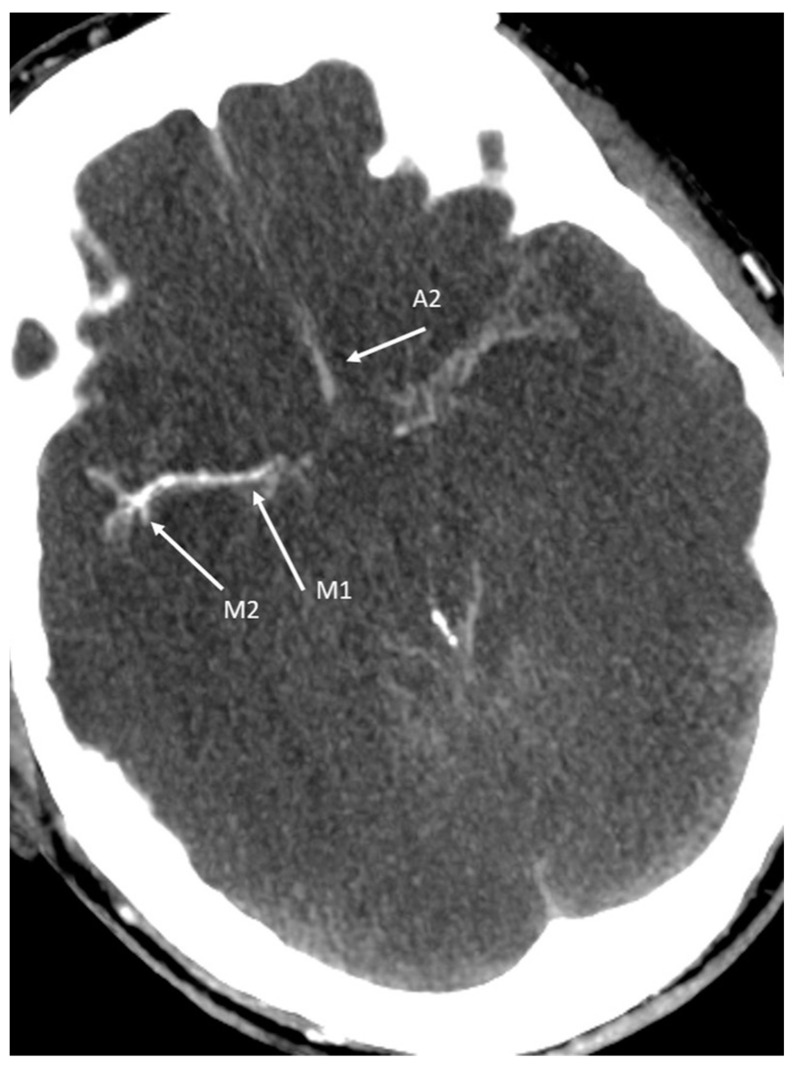
53-year-old female patient with comatose state (GCS 3) and with clinical signs of brain death after propofol overdosage with suicidal intent. Stasis filling of the anterior cerebral artery (A2 segment) and the middle cerebral artery (M1/M2 segment). Not shown: lack of contrast of the posterior circulation (basilar artery, posterior cerebral artery, M4 segments and deep veins—ICV, GCV). The presented findings don’t preclude the diagnosis of brain death.

The most sensitive scoring system appears to be the 4-point scale with a sensitivity of 88% [43]. The reason for the development of the 4-point score is the contrasting of the basilar artery and proximal parts of the middle, anterior and posterior cerebral artery, often observed in the 10-point score, which can occur despite the presence of BD (“stasis filling”) [38]. The term “stasis filling”, defined as delayed, weak, and persistent opacification of proximal segments of the cerebral arteries not reaching cortical branches (no opacification of the cortical branches or venous outflow), was first introduced by Kricheff et al. [45] and is commonly observed in angiographic studies. This phenomenon is a consequence of raised intracranial pressure (ICP) and high cerebrovascular resistance (CVR). Cessation of capillary circulation is consistent with CCA while proximal arterial segments are still patent. An example of stasis filling is shown in Figure 2.

**Figure 3:**
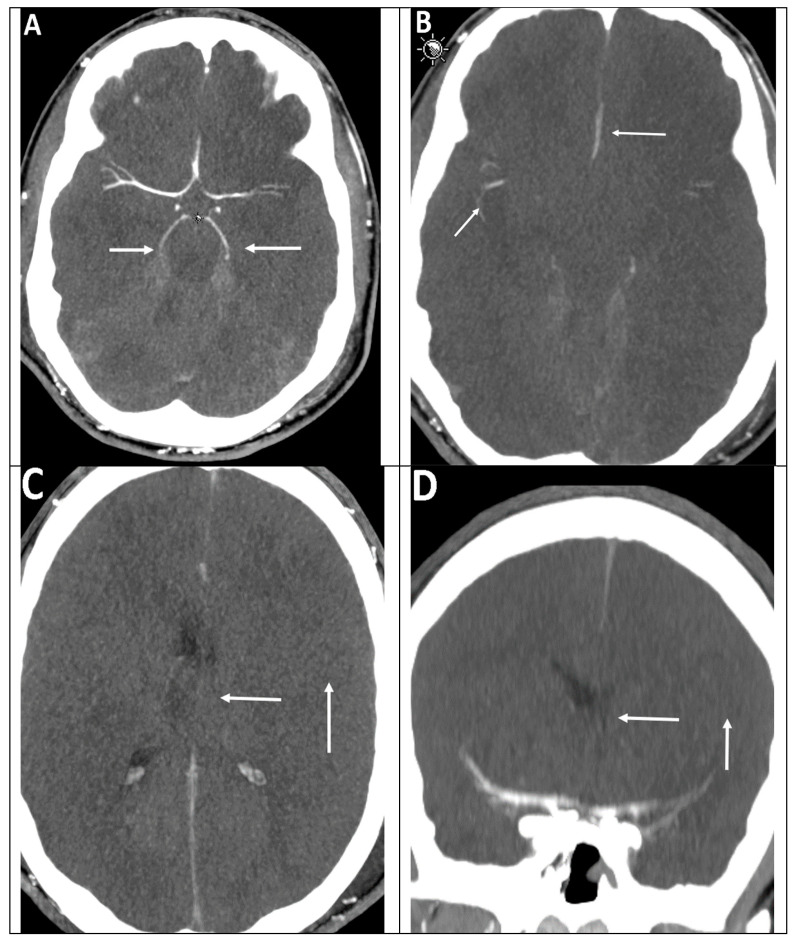
(**A**) Stasis filling in the proximal segments of ACA (A1, A2), MCA (M1, M2), and PCA (P1, P2, arrows). (**B**) Stasis filling also of the M3 segments of the MCA and the A3 segments of the ACA (arrows). The criteria of cerebral perfusion arrest are not fulfilled according to 7-point and 10-point CTA score. Axial (**C**) and coronal (**D**) CTA images demonstrating the lack of contrast filling of the M4 segments of the MCA and the ICV (arrows) consistent with the diagnosis of cerebral circulatory arrest according to the 4-point scale.

The discrepancy in the relevance of the various scoring systems is shown by the clinical example of a 15-year-old male comatose patient (with clinical signs of brain death) after near-drowning (Figure 3).

- Compared to DSA, Zampakis et al. [44] found no statistically significant difference between all CTA -4-point scores and in general, the 4-point CTA scoring system has been accepted as the most reliable scoring among other CTA scoring systems in the diagnosis of BD. However, some challenges still exist [46]. For completeness, it should be noted that there are different versions of the 4-point score [44]: the 4-point scale introduced by Frampas et al. [38] (the so-called CTA-F (by this score only the M4 branches of the middle cerebral artery and the internal cerebral vein are assessed);
- The revised 4-point scale suggested by Nunes and co-workers [47] (the so-called CTA-MF) and
- The prior revised venous 4-point scale, proposed by Marchand and colleagues [48] (the so-called CTA-M).

There are data that support the assumption that CTA-M could increase the sensitivity, but the evaluation of the superior petrosal vein opacification may be challenging (caused, e.g., by brainstem herniation or subarachnoid hemorrhage around the brainstem) and it would not be as practical as that of the CTA-F [46]. A cursory overview of the differences between the various CTA-scoring systems is summarized in Table 2.

## 4. The Role of Computed Tomography Perfusion (CTP) as Adjunct in Diagnosing BD

As shown above, using CTA to confirm BD may induce specific diagnostic challenges, such as the potential persistence of blood flow in patients with a clinically confirmed diagnosis of BD [36]. The diagnostic confusion can be induced by the preserved filling of the cortical branches (e.g., middle cerebral artery (MCA), the internal cerebral veins (ICV), or both. This phenomenon has been observed in about 15% of brain-dead patients [49]; however, the causal stasis filling, does not necessarily preclude the diagnosis of BD [36].

CTP is an advanced CT scan technique that provides information on cerebro-vascular dynamics and state. By using contrast agents and special postprocessing software, perfusion is detected even in small vessels such as arterioles, capillaries, and venules [50]. This imaging technique can help in the calculation of cerebral blood flow (CBF) and cerebral blood volume (CBV). Shankar & Vandorpe [50] studied CTP derived from patients clinically suspected of BD and showed that CTP could be a valuable ancillary tool (with 100% sensitivity) in the early detection of brain death. Sawicki et al. [30] tested the reliability and diagnostic accuracy of CTP over CTA in determining BD. For whole-brain CTP they also showed a sensitivity of 100% to confirm the diagnosis of BD. In brain-dead patients, CTP results revealed CBF 0.00 – 9.98 mL/100g/min and CBV 0.00 – 0.99 mL/100g, and were thus interpreted as positive in all patients. The difference between values of CBF and CBV in the brain-dead and non-brain-dead groups was statistically significant. Similar sensitivity of CTP was reported by MacDonald et al. [31]. Accordingly, the whole-brain CTP might be decisive in some cases of inconclusive CTA [29]. However, variations in the quantitative analysis by using different postprocessing methods are potential limitations of CTP. Moreover, only qualitative analyses rather than quantitative analyses are needed for diagnosing BD. With the increased coverage provided by newer multisection CT scanners and improved experience the actual limitations should be reduced [50]. However, we agree with other authors [29,51] that for further research advancement, a uniform and internationally accepted benchmark for the necessary large series of prospective clinical trials evaluating various topics of CTA and CTP methods (and also other ancillary tests) should be established.

## 5. General Considerations to Secure the Informative Value of CTA/CTP

In order to provide adequate preconditions for relevant CTA/CTP-findings, special attention must be focused on the hemodynamic stability during the examination procedure for the evaluation of cerebral blood perfusion for BD-determination. A mean arterial blood pressure of >80 mm Hg [36] or a systolic blood pressure of at least 100 mm Hg [26] for the examination period is required in studies or national recommendations, respectively. In addition, CT angiograms are technically adequate with opacification of both superficial temporal arteries in the arterial phase indicating sufficient intravenous bolus injection of the contrast medium and therefore as evidence of the arrival of the intravascular contrast medium in the area of the brain-supplying arteries [26]. 

Further, proper timing based on time elapse after the appearance of brain stem areflexia and/or Doppler-pre-test might significantly reduce preterm examinations and significantly increase sensitivity of CTA in CCA diagnosing procedures. The use of French criteria based on 4-point score showed that short time between clinical brain death and CTA leads to higher number of inconclusive results (low sensitivity) and it is postulated that time delay >6 h provides sensitivity of 92% [52]. Similar tendency was reported by Welschehold et al. [39]. This clearly points out that proper timing (which should be a pragmatic, not unnecessarily diagnosis delaying investigation frame) is a crucial factor determining CTA sensitivity regardless of protocol used and that minimal time span should be recommended in national guidelines. 

## 6. Cerebral Perfusion Test as Diagnosing Option in False-Negative Ventilation Patterns

The presence of apnea is a critical clinical sign for the diagnosis of BD, but ventilator autotriggering (VAT) can mimic a central respiratory drive by triggering the respirator even though apnea is present and all cephalic reflexes are absent. This can lead to uncertainty in BD determination and may result in a time delay or even cancellation of the BD diagnostic process [17,18,19]. This phenomenon, sometimes underestimated in its consequences, is more common in newer-generation ventilators because they have internal programming that allows assisted breaths to ensure the patient’s oxygenation during prolonged periods of apnea, regardless of the trigger setting selected. The extrapolated prevalence is about 10–12%. The possible extrinsic (e.g., various changes in the entire ventilatory circuit like leaks or excessive condensation as well as inappropriate trigger mode and sensitivity) and intrinsic causes (e.g., cardiogenic respiratory oscillations) as well as diagnostic measures and approaches to terminate VAT (e.g., change from flow trigger mechanism to pressure trigger mode) should be considered [17]. Because the clinical and electrophysiological signs of BD in the presence of VAT are contradictory to the displayed respiratory parameters and respirator functionality, this phenomenon may lead to confusion among critical care staff. The fact that VAT is not widely known further exacerbates the situation. VAT is mentioned in only 3 of 15 selected European national guidelines for the determination of BD, and the procedure for detecting and eliminating VAT is explained only in two of the guidelines [17]. As ventilatory conditions are not trivial in every case and uncertainties may persist, cerebral perfusion testing [53] and/or interdisciplinary consultation should be considered for diagnosis and documentation.

## 7. Cerebral Perfusion Tests in Case of Possible False-Positive Findings Caused by Central Nervous System (CNS) Depressant Drugs 

Current BD diagnostic recommendations agree that brainstem function tests should not be performed under the effect of CNS depressant drugs and neuromuscular blockers [25] and the presence of these confounders has to be excluded a priori since they may invalidate the clinical examination results. The conventional diagnostic procedure ranges from assessment of drug history, drug screening, or calculation of drug clearance to demonstration that plasma levels of drugs are below the therapeutic threshold if present [15,54]. There is a country-specific inconsistency regarding whether the detection of absent cerebral blood flow by CTA or TCD/CCDS is optional [55] or, in this specific case, mandatory [26]. However, in daily practice, the most frequent iatrogenic cause of delay in the diagnosis of BD on the basis of clinical criteria is treatment with CNS depressants such as barbiturates, opioids, propofol or benzodiazepines (BZDPs) [56]. Based on our own retrospective unpublished data, we focus here on benzodiazepine (BZDP) medication issues. In 32 adult patients suspected of brain death, CTA was performed in 6 cases (with evidence of CCA). In all cases, the indication for CTA was treatment with central depressant drugs, with benzodiazepine administration present in 5 cases. This group of pharmaceuticals, especially midazolam, is still widely used for sedation in intensive care patients. However, not only the expected but also the unintended depressant effects of BZDPs on the CNS must be considered in diagnosing BD. The various causes of potential pharmacodynamic and pharmacokinetic conditions during intensive care treatment with unintended oversedation with BZDPs ranging from, e.g., altered pharmacokinetic and pharmacodynamic properties in (elderly) intensive care patients [57], drug interactions [58,59], redistribution of BZDPs after reperfusion of previously cooled tissues after therapeutic hypothermia [16] up to the possible accumulation of CNS-active conjugates of midazolam in renal (or hepatic) dysfunction although plasma levels of parent drug may even decrease [60,61] can be summarized. From a practical perspective, antagonizing BZDPs is intended to meet the requirements of BD diagnosis in a timely manner, thereby minimizing organ loss due to procedural delays. 

However, there is a controversial discussion regarding the possible severe side effects of flumazenil in deeply comatose patients. The unconfirmed efficacy (wide effective dose range [62]), short effect, low predictive value [16], the possible severe side effects (further increase of ICP in patients with elevated intracranial pressure [63], induction of cerebral convulsions [64] and severe cardiocirculatory side effects [65,66] should be critically considered. The inconsistency of recommendations on antagonism in various national guidelines, even in Europe (e.g., recommended for the UK [67] vs. not recommended for Spain [68], as well as the medico-legal implications regarding restricting indications by drug information [69] are factors worth of consideration. In some publications that either favored antagonist administration [55,70,71,72,73,74,75,76] or expressed caution with regard to patient safety or doubts about the efficacy of the drug in brain death [16,70,77], we found no references in the respective articles to previous clinical studies in patients in whom brain death was suspected (e.g., comparison with a gold standard such as a cerebral perfusion test) or to animal studies in a brain death model. This represents a gap in the literature that requires further selective research. Therefore, the documentation of cerebral circulatory arrest (and, if necessary, its repetition or, if uncertainty persists, the laboratory chemical-toxicological examination taking standardized methods for the qualitative and quantitative analysis of drugs into account [78]) seems appropriate from the point of view of patient safety and the plausibility of the test maneuvers in the current state of publications.

## 8. CTA and CTP as Contents of an Expert-System Based Training Tool for Diagnosing BD

The titles of the articles “Why is diagnosing brain death so confusing?” [79] or “Determination of brain death—no room for error” [80] point to the problems of the diagnostic procedure that is quite demanding in individual cases and should be understood as particular warnings. Clinicians and students may have limited opportunities to perform all the steps of the whole clinical process in diagnosing BD. There is indeed a lack of bed-side postgraduate and undergraduate training in real patients to acquire the necessary skills since the diagnosis of BD is rarely made in everyday practice. This deficit is accentuated outside of a central or specific intensive care unit (ICU). Furthermore, the topic of “death” in general is highly underrepresented in medical education [81]. These factors are contributory causes that should not be underestimated for the increasing gap between the number of donor organs required and those actually available worldwide [82]. Since deficiencies in knowledge regarding (i) various confounders (due to different causes) influencing adequate classification of clinical manifestations, (ii) the procedure of clinical testing, and (iii) the technical-methodological approach and interpretation of ancillary diagnostic techniques may delay or even prevent the determination of BD in individual cases. With this in mind, the e-learning tool BRAINDEXweb (developed by the Institute for Medical Informatics, Statistics and Documentation at Medical University of Graz, Austria) also addresses essential considerations related to CTA/CTP.

BRAINDEXweb is a training tool based on a rule-based expert system and implemented as a web application, which guides the user in a dialogue through all diagnostic steps relevant for brain death assessment when the condition of the (fictitious or real) patient appears to be brain dead. BRAINDEXweb is a technical advancement of the earlier IT-based documentation system on deeply comatose intensive care patients [83,84]. The selection and sequence of questions is adapted to the modality a medical expert would proceed, according to the Austrian Recommendations for the Performance of Brain Death Diagnostics in the Case of Planned Organ Removal [26]. The inference engine (i.e., the software component that makes decisions based on the facts and rules contained in the knowledge base) operates as a backward chaining rule engine and attempts to prove the three hypotheses (“goals”) state of the patient is “compatible with brain death”, “not compatible with brain death”, and “not assessable”. In addition, some supporting features are available, such as background information on the currently asked question, a lexicon specific to BD diagnostics, a bibliography, and - for the area of spinal reflexes - an additional program with an interactive schematic homunculus, further explanatory texts and videos. If there is insufficient or seemingly contradictory input, or if planned examinations could harm the patient (e.g., apnea testing in conditions at risk of severe hypoxemia), the system issues warnings. As a result, BRAINDEXweb generates a protocol containing all the information provided, an assessment of the patient’s status according to the recommendations on BD diagnosis [26], and explanations of this assessment. The protocol is largely in line with the recommendations for BD diagnosis but, in some areas, goes far beyond the scope and level of detail of the official recommendations (e.g., CTA, CTP, ventilator autotriggering etc.).

### Example of BRAINDEXweb-Workflow in the Context with False-Negative CTA-Signs Caused by Decreased Intracranial Pressure (ICP) in Suspected BD

**Figure 4:**
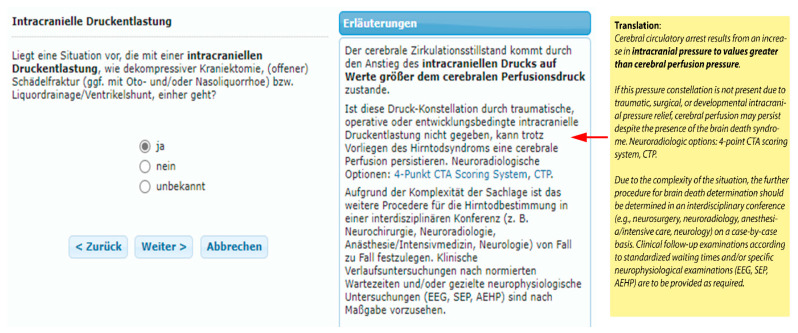
BRAINDEXweb-Screenshot of the immediate consequence by the system when no CCA is diagnosed in clinically suspected BD. The system reacts with the question for the existence of an intracranial decompression caused by craniectomy, trauma, oto-naso-liquorrhoe or liquor-drainage (left box). Notes for pathophysiology, additive neuroradiological options (links to CTA, CTA-Scoring, CTP in the lexicon) and potential clinical or electrophysiological procedures are presented in the comments of the middle box of the figure (the user interface is in German; for an English translation of the explanatory text indicated by the red arrow, see the box on the right).

Using a fictional case of suspected BD (with clinical signs of complete brainstem areflexia and isoelectric EEG), it is shown here how BRAINDEXweb would react if no cerebral blood flow arrest could be detected by CTA. The system poses the control question as to whether intracranial pressure decrease is present (Figure 4). In this context notes for pathophysiology, optional neuroradiological procedure and potential clinical or electrophysiological measures are given. There are also links to the dictionary with specific comments on CTA, CTA-scoring and CTP.

In summary, it should be noted at this point that the problems surrounding CTA should also be included as a content of a general education system for intensivists, who ultimately have to make the decision of BD. 

## 9. Cerebral Perfusion Test as Diagnosing Option in Case of False-Negative Movement Patterns

**Figure 5:**
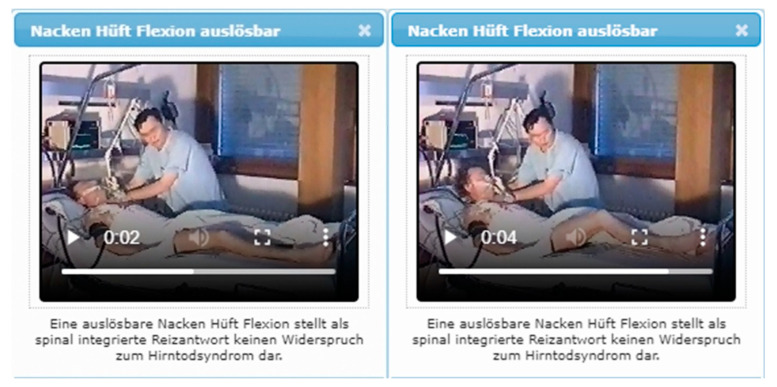
Screenshot of a video clip of BRAINDEXweb regarding neck-hip flexion before reflex triggering (**left**) and during reflex release (**right**) in a patient with clinically signs of BD (apnea, loss of all brain stem reflexes, isoelectric electroencephalogram).

A cause of false-negative BD-evaluation in deeply comatose patients with Glasgow Coma Scale Score (GCS) 3 and complete loss of all cephalic reflexes and proven apnea may be due to one or more spinal automatisms. These spinal integrated responses/movements may occur stimulus provoked or spontaneously after a period of approximately 2–48 h or more (after the disappearance of the spinal shock) and in some cases are qualitatively altered in brain death, i.e., the stimulus responses may be delayed, slowed in sequence, fatigued, or there may be hyperexcitability. The areas of reflex triggering do not remain coupled to the corresponding dermatomes, but may also transgress them [20,22]. However, when clinical criteria for brain death are met, the prompt recognition of spinal associated movements can reduce uncertainty and unnecessarily prolonged treatment [85]. (For an illustration of possible spinal reflexes see the screenshot of one of the corresponding video clips of BRAINDEXweb displayed in Figure 5). 

In case of persistence of doubt the consultation of an experienced neurologist/neurosurgeon and/or confirmatory testing such as the examination of the cerebral circulation arrest [86,87] and/or the registration of cerebral bioelectrical signals (e.g., evoked potentials [88], electroencephalogram [89] are confirmatory options reported in the literature that can be in accordance with the respective national guidelines for BD-diagnosing.

## 10. Conclusions

CTA has the potential for the future to be the test of choice for the diagnosis of cerebral perfusion arrest. This applies to both cases with possible false-positive or false-negative clinical findings. According to the current state of the literature, one of the essential factors for valid results in brain death determination is the appropriate scoring selection whereby the 4-point CTA scoring system has been accepted as the most reliable score. The whole-brain CTP could be crucial in some cases of an inconclusive CTA. Appropriate timing and/or Doppler pre-testing could significantly reduce the number of possible premature examinations and significantly increase the sensitivity of CTA in diagnosing CCA. The inconsistency of study designs on ancillary tests makes further specific studies using a uniform and internationally accepted benchmark for large series of comparative prospective clinical trials necessary. Since the responsibility of BD determination lies with the treating intensive care physicians of various medical disciplines (ideally interdisciplinary), the options and possible limitations of CTA should also be communicated via innovative electronic training/education tools.

## Figures and Tables

**Table 1:** Ancillary tests recommended by various guidelines for brain death diagnosing [26,27,28].

Target Components	Ancillary Tests	Advantages	Disadvantages/Limitations
Cerebral bioelectrical activity	Electroencephalogram (EEG)	Bedside applicable Non-invasive No contrast media required	Interference susceptibility due to electrically ICU-operating-equipment or biogenic artifacts (false-negatives) Interaction with sedative acting substances/marked hypothermia (false-positives)
	Evoked potentials: Somatory evoked potentials (SEP), Brainstem auditory evoked potentials (BAEP)	Bedside applicable Non-invasive No contrast media required More stable against sedatives than EEG	Interference susceptibility due to electrically ICU-operating-equipment (false-negatives) Interaction with sensory conduction disturbances/abolished stimuli perception (false-positives): - SEP: e.g., median nerve lesion, axillary plexus lesion, cervical spinal lesion - BAEP: e.g., pre-existing deafness, petrosal bone fracture, ototoxic drugs
Cerebral Circulation	Transcranial Doppler sonography (TCD)/Color-coded duplex sonography (CCDS)	Bedside applicable Non-invasive No interaction with sedative substances No contrast media required	Absence of temporal insonation window in 10–20% Operator-dependent False-negatives in reduced ICP (traumatic, neurosurgical, post-anoxic)
	Digital subtraction angiography (DSA)	No interaction with sedative substances	Invasive (performed with a catheter tip in the aortic arch and contrast injection into each of the four arteries supplying the brain) No spatial resolution to distinguish the blood flow in the different parts of the brain such as brainstem Operator-dependent Time-consuming Potential risk to contrast medium False-negatives in reduced ICP (traumatic, neurosurgical, post-anoxic) Not bedside feasible
	Computer tomography angiography (CTA)	No interaction with sedative substances Scantly invasive Technically uncomplicated Not-time consuming Widespread availability	False-negatives in reduced ICP (traumatic, neurosurgical, post-anoxic) Actual inconsistency in scoring systems Inconsistent benchmark for clinical trials evaluating the method Only anatomic information Iodinated contrast required Not bedside feasible
	Computed tomography perfusion (CTP)	No interaction with sedative substances Scantly invasive Comparatively highest sensitivity and specificity Information on brain function and anatomy Demonstration of isolated brainstem death Feasibility with standard CT scanners Relatively small amount of contrast agent necessary Less operator dependent	Post-processing software of acquired data necessary Low comparability of studies by use of different postprocessing methods Gap of large prospective series Inconsistent benchmark for clinical trials evaluating the method Not bedside feasible
	Radionuclide cerebral blood perfusion imaging	Scantly invasive Reliable and reproducible data (“empty skull sign”/“hollow skull phenomenon”) No uptake of the radiopharmaceutical due to medication or metabolism No deleterious effects of radiopharmaceuticals on potential donor organs Not operator dependent Information on intracranial blood flow and brain parenchyma activity	False-negatives in reduced ICP (traumatic, neurosurgical, post-anoxic) Inconsistent benchmark for clinical trials evaluating the method Poor spatial resolution (e.g., posterior fossa may be difficult to visualize isolated brainstem activity) Associated time delay Infrastructure of nuclear medicine department and specific experience for interpretation of test results necessary Reduced device availability (especially portable gamma head camera) Expensive radioactive tracer Improper labelling of brain-specific radiopharmaceuticals or injection of wrong radiopharmaceuticals can result in false-positive results Inconsistency of reported tracers (e.g., brain non-specific agents vs. brain specific agents) Blood drainage into the superior sagittal sinus causes false-negatives Confirmation of correct IV injection may need additional images of other organs (e.g., liver) demonstrating uptake for exclusion of false-positives
	Magnetic resonance imaging (MRI)	Reliable high-resolution imaging No contrast media required Non-invasive Accurate in identifying structural abnormalities	Length of the scan time ICU-patients may have several contraindications to MRI (e.g., surgically or traumatically inserted ferromagnetic materials, electronic implants) Combination of multiple criteria for diagnosing BD (not generally accepted in imaging guidelines for BD) SWI findings not specific due to false-positive findings.

ICP = intracranial pressure, ICU = intensive care unit, SWI = susceptibility-weighted imaging, IV = intravenous, BD = brain death

**Table 2:** CTA- scoring systems for determination of brain death. MCA: middle cerebral artery, ACA: anterior cerebral artery, PCA: posterior cerebral artery, BA: basilar artery, ICV: internal cerebral vein, GCV: great cerebral vein, SPV: superior petrosal vein. (a) Only in arterial phase. (b) Only in venous phase. CTA-F: 4-point score introduced by Frampas et al. [38]; CTA-M: 4-point score introduced by Marchand et al. [48]; CTA-MF: revised CTA-F introduced by Nunes et al. [47].

CTA-Scoring Systems	Cerebral Arterial and Venous Vessels (Quantity of Assessed Vessels)							Total Number of Assessed Vessels
10-point scoring	MCA-M4: (2)	ACA-A3: (2)	PCA-P2: (2)	BA: (1)	ICV: (2)	GCV: (1)	(-)	10
7-point scoring	MCA-M4: (2)	ACA-A3: (2)	(-)	(-)	ICV: (2)	GCV: (1)	(-)	7
4-point scoring (CTA-F)	MCA-M4: (2)	(-)	(-)	(-)	ICV: (2)	(-)	(-)	4
4-point scoring (CTA-M)	(-)	(-)	(-)	(-)	(-)	GCV: (2)	SPV: (2)	4
4-point scoring (CTA-MF)	MCA-M4: (2, a)				ICV: (2, b)			4

## Data Availability

The software described in this paper, developed in the framework of this BRAINDEXweb project, is defined as a proprietary deliverable. Therefore, the source code of this software cannot be made open source or shared through open access.
